# Efficacy and Safety of Calcifediol in Young Adults with Vitamin D Deficiency: A Phase I, Multicentre, Clinical Trial—POSCAL Study

**DOI:** 10.3390/nu16020306

**Published:** 2024-01-19

**Authors:** Pedro Guerra López, Mikel Urroz Elizalde, Noelia Vega-Gil, Blanca Sánchez Santiago, Iñaki Zorrilla Martínez, Mario Jiménez-Mercado, Esteban Jódar, Araitz Landeta Manzano, Cristina Campo Hoyos, Jesús Frías Iniesta

**Affiliations:** 1 Clinical Trials Unit, Pharmacology Department, Universidad Autónoma de Madrid, 28049 Madrid, Spain; pedro.guerra@uam.es (P.G.L.); mikel.urroz@salud.madrid.org (M.U.E.); jesus.frias@uam.es (J.F.I.); 2 Clinical Pharmacology Service, Hospital Universitario La Paz, 28049 Madrid, Spain; 3 Valdecilla Clinical Trials Unit, Hospital Universitario Marqués de Valdecilla-IDIVAL, 39008 Santander, Spain; noelia.vega@scsalud.es (N.V.-G.); mblanca.sanchezs@scsalud.es (B.S.S.); 4 Clinical Trials Unit, IIS BIOARABA, 01009 Vitoria-Gasteiz, Spain; inaki.zorrillamartinez@osakidetza.eus (I.Z.M.); marioernesto.jimenezmercado2@osakidetza.eus (M.J.-M.); 5 Mental Health and Childhood Research Group, IIS BIOARABA, 01009 Vitoria-Gasteiz, Spain; 6 Psychiatry Department, Araba University Hospital, 01009 Vitoria-Gasteiz, Spain; 7 Department of Neurosciences, University of the Basque Country UPV/EHU, 01006 Vitoria-Gasteiz, Spain; 8 CIBER of Mental Health (CIBERSAM), Institute of Health Carlos III, 28029 Madrid, Spain; 9 Department of Endocrinology and Nutrition, Quirónsalud Madrid University Hospital, 28233 Madrid, Spain; esteban.jodar@quironsalud.es; 10 School of Health Sciences, European University of Madrid, 28670 Madrid, Spain; 11 Medical Affairs, FAES FARMA S.A., 48940 Leioa, Spain; alandeta@faes.es

**Keywords:** 25-hydroxyvitamin D3, calcifediol, clinical trial, vitamin D deficiency, young adults

## Abstract

Vitamin D deficiency is highly prevalent, and recent evidence suggests a possible association between vitamin D deficiency and various health conditions. The aim of this study was to assess monthly calcifediol treatments for vitamin D deficiency (or biweekly, if the deficiency was severe) in a young adult population with no associated comorbidities. This multicentre phase I trial started with a four month open-label treatment phase (TP) that included 101 participants (65% women with mean age 29.8 years). Eighty-two percent of the subjects (79/96) achieved 25(OH)D levels within the target range (20–60 ng/mL) by the end of the TP, and they were subsequently randomised and subjected to a double-blind, placebo-controlled, five month follow-up phase (FP). At the end of the FP, 89% of participants maintained vitamin D levels of >20 ng/mL with calcifediol, versus 49% with placebo (*p* < 0.001). Subjects receiving monthly calcifediol during both phases (*n* = 32) maintained 25(OH)D levels >20 ng/mL, whereas those on the placebo during the FP (*n* = 38) exhibited deficiency levels of 25(OH)D by the end of the study. No clinically relevant changes in bone metabolism parameters or toxic 25(OH)D levels were observed, and no serious adverse events were reported throughout the study. Calcifediol is a safe and effective treatment for vitamin D deficiency in the young adult population, but long-term use may be required to sustain optimal 25(OH)D levels.

## 1. Introduction

Vitamin D deficiency is a widespread condition, affecting around one billion individuals across the globe [1]. Ever since the discovery of the importance of Vitamin D in treating rickets in 1922 [2], a substantial body of research has emerged. This research not only underscores the well-established connection between vitamin D and bone health, but it also hints at a potential link between low 25-hydroxyvitamin D (25(OH)D) levels and several acute and chronic illnesses [3,4,5]. Remarkably, several experts and researchers have gone so far as to assert that vitamin D should not be viewed as a typical vitamin, but rather as a complex endocrine or hormonal system, known as the vitamin D endocrine system [6].

Although vitamin D can be acquired from different food sources, such as oily fish (e.g., cod, salmon, etc.), eggs, milk, and mushrooms, its main source is endogenous synthesis in the skin. Solar ultraviolet-B radiation converts 7-dehydrocholesterol into previtamin D_3_, which is then rapidly converted into vitamin D_3_ (cholecalciferol). This molecule needs to be metabolised in the liver into 25(OH)D_3_ (also called calcifediol or calcidiol). Calcifediol undergoes a second hydroxylation via the enzyme CYP27B1, which produces the final active molecule, 1,25(OH)_2_D_3_ (calcitriol). This step mainly occurs in the kidneys, although CYP27B1 has also been found in various other organs and tissues [7].

It is widely accepted that 25(OH)D is a biomarker of vitamin D status. However, there is controversy surrounding optimal 25(OH)D levels, with some societies such as the National Academy of Medicine (formerly known as the Institute of Medicine) setting a threshold of 20 ng/mL, whereas others such as the Endocrine Society set it to 30 ng/mL [8]. Nevertheless, most of the main scientific societies consider 25(OH)D levels <20 ng/mL to be an indicator that deficiency is present [9].

The first step in the management of hypovitaminosis D in the general population is to try to increase sun exposure and the intake of foods containing vitamin D. However, these measures are insufficient in many cases, and doctors must prescribe pharmacological supplementation. In these patients, hypovitaminosis D can be treated with ergocalciferol (D_2_), cholecalciferol (D_3_), or calcifediol (25(OH)D_3_); cholecalciferol is the most widely used.

Calcifediol has been readily accessible and prescribed extensively in Spain for over four decades, but it has only recently gained recognition in global recommendations and guidelines, since it has become available in other nations. Some studies have shown that calcifediol is more potent and rapid than cholecalciferol in correcting 25(OH)D levels [10,11]. The heterogeneity in the data from such studies (small, local studies, elderly population, other comorbidities as confounding factors) provides limited information and does not allow for more general extrapolations to be made.

Considering all of the above, this study was designed to collect data on the behaviour of calcifediol in the treatment of vitamin D deficiency in a population of young adults with no other associated comorbidities, with two different dose regimens commonly used in clinical practice.

## 2. Materials and Methods

### 2.1. Design

This multicentre phase I clinical trial was conducted at three clinical sites in Spain, in accordance with the ICH Harmonized Tripartite Guidelines for Good Clinical Practice, applicable regulatory requirements, and the latest updated version of the Declaration of Helsinki (2013). It was approved by the Ethical Committee for Research with Medicines (CEIm) at the La Paz University Hospital (Madrid, Spain), (HULP code) 5610, 23 July 2020.

**Figure 1:**
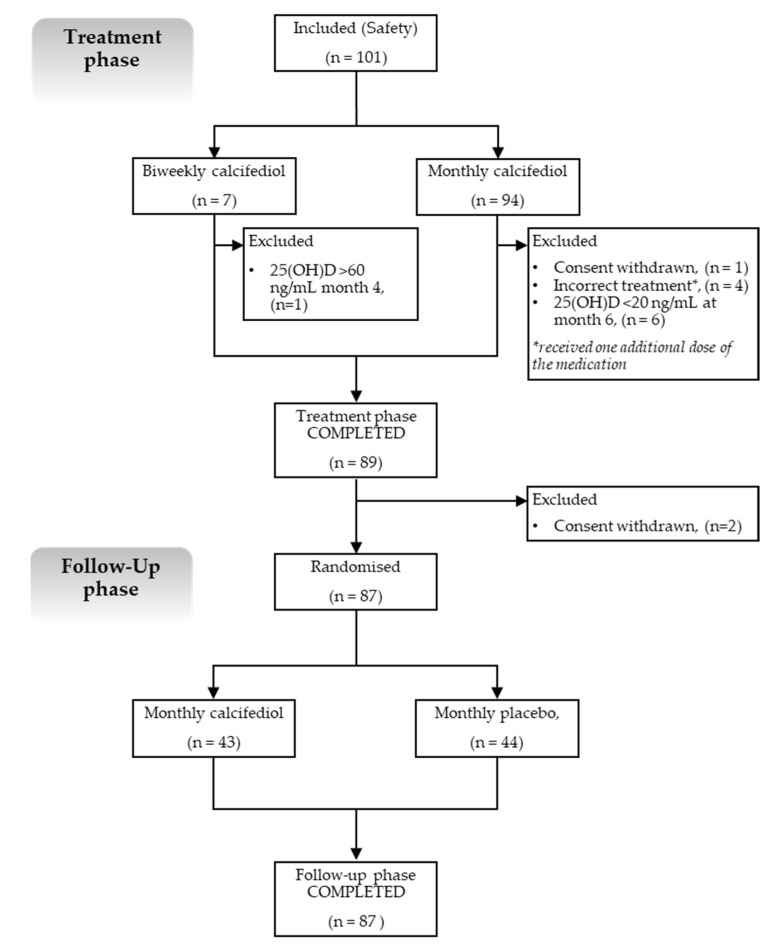
Flow diagram of the participants and study design.

The study comprised two phases, as follows: an open-label treatment phase and a double-blind, placebo-controlled, follow-up phase (Figure 1).

In the treatment phase, subjects with vitamin D deficiency (defined when 25(OH)D levels were <20 ng/mL) received calcifediol (0.266 mg soft gelatine capsules) either monthly or every two weeks, depending on their baseline 25(OH)D levels. Subjects with mild to moderate deficiency (25(OH)D levels from 10 to <20 ng/mL) received monthly treatment, whereas those with severe deficiency (25(OH)D levels <10 ng/mL) received biweekly treatment.

All subjects received treatment with calcifediol for 4 months, after which, 25(OH)D levels were measured (in month 4). If subjects had reached the 25(OH)D target range (defined as 20–60 ng/mL), they proceeded to the follow-up phase.

If they did not reach the target range, they continued calcifediol treatment for an additional 2 months, after which, 25(OH)D levels were measured again (in month 6). If levels were within the target range by month 6, the subjects entered the follow-up phase. If 25(OH)D levels persisted below 20 ng/mL, subjects were withdrawn from the study; if levels were above 60 ng/mL, subjects stopped receiving calcifediol treatment and were followed until the end of the study for safety reasons.

The follow-up phase included subjects who had already reached target 25(OH)D levels, who were randomised to receive masked monthly treatments with calcifediol 0.266 mg or placebo for 5 months. At the end of this phase, the 25(OH)D levels were measured again.

Hence, the overall study duration for the participating subjects ranged between 9 and 11 months, depending on their 25(OH)D levels by month 4.

The treatment phase was designed to address the primary objective of the study, as follows: to determine the percentage of participants with vitamin D deficiency who achieved the pre-specified target range (plasma 25(OH)D levels between 20–60 ng/mL) after 4 months of treatment.

The follow-up phase (or maintenance phase) was then designed to assess the need to continue (or not) with calcifediol treatment in order to maintain 25(OH)D levels within the target range; then, the final 25(OH)D levels of the participant groups treated with calcifediol and placebo were compared.

Calcifediol safety and tolerability profiles were evaluated throughout the study by collecting and analysing the number and seriousness of adverse events (AEs) reported by study participants. In addition, bone metabolism parameters were also specifically monitored and recorded.

### 2.2. Participants

Eligible participants included adults aged 18–50 years with vitamin D deficiency (defined as plasma 25(OH)D levels <20 ng/mL) with no other associated relevant conditions. Subjects diagnosed with any serious pathology, such as liver failure, congestive heart failure, or renal impairment, were excluded from participation. They were also excluded if they met any of the following criteria: primary hyperparathyroidism, hypothyroidism, hypercalcaemia, or a body mass index <18.5 or >30 kg/m^2^. Subjects taking any medication that could interfere with vitamin D metabolism (e.g., thiazide diuretics, hydrochlorothiazide, parathyroid hormone, bisphosphonates, phenobarbital, phenytoin, etc.), or who tested positive for illicit drugs or ethanol in urine, were also ineligible. Those receiving current treatment with any vitamin D analogues, vitamin D supplements, or calcium supplements needed to comply with a washout period prior to entering the study.

Written informed consent was obtained from all participants prior to participation in the study.

### 2.3. Data Collection

The data collection period was from August 2020 to April 2022. The participating sites were the three Clinical Trials Units at the Pharmacology Dpt. Universidad Autónoma de Madrid (Madrid), the Hospital Universitario Marqués de Valdecilla (Santander), and the Hospital Universitario Araba (Vitoria) (Spain).

The demographic and clinical characteristics of the participants were collected at baseline before starting treatment. During the first visit, vital signs were recorded, as well as data from the physical examination, ECG, and blood and urine analyses. Plasma 25(OH)D concentrations were determined in the laboratory of each local hospital via immunoassay at different time points in the study, as follows: baseline, month 1, month 4, month 6 (if applicable, as described previously), and five months after the start of the follow-up phase (in month 9 or 11). Additionally, relevant bone metabolism parameters, such as serum calcium, intact parathormone (PTH), phosphate, albumin, and alkaline phosphatase (ALP) were monitored at the same time.

Safety endpoints included the collection of AEs, especially serious and treatment-related AEs, as well as patients withdrawn from the study due to safety concerns. During the study, an electronic diary was used to record the date of the study, medication intake, any AE that occurred during the study, and the medication administered to treat the AE, if applicable. All collected AEs were coded in accordance with the Medical Dictionary for Regulatory Activities (MedDRA) version 25.0.

### 2.4. Statistical Methods

The sample size was calculated based on the estimated proportion of patients who reached the target range for 25(OH)D levels at month 4 through a normal asymptotic two-tailed 95% confidence interval, assuming that the proportion of the population who reached this target range and who met the criteria was 50% (at least 50–60% of subjects treated with calcifediol for 4 months could achieve 25(OH)D levels within the target range). Taking all of the above into consideration, 100 participants were deemed eligible for inclusion in the study.

A randomisation list was created for the follow-up phase of the study only. The treatments were randomly assigned using the EPIDAT 4.2 program [12]. Subjects were blindly allocated to one of the monthly treatment arms of the maintenance phase (calcifediol or placebo).

Different population sets were defined for the safety and efficacy analysis. The safety population included all study participants who had been exposed to at least one dose of the study drug. The efficacy analysis was conducted separately for both phases of the study. During the treatment phase, the population for the efficacy analysis included all subjects who completed the treatment phase, with determination of at least baseline and 4 month 25(OH)D levels, as long as the subjects had correctly taken the study medication according to protocol (Full Analysis Set [FAS]). During the maintenance phase, the efficacy analysis was conducted in the population that comprised all participants who started the follow-up phase with 25(OH)D levels that were measured during the final visit (month 9 or 11), after they took the study medication correctly, in accordance with protocol (Per Protocol Population [PP]).

Quantitative variables were summarised using mean and standard deviation (SD), and categorical variables were presented in terms of frequency and percentages. ANOVA was performed repeatedly in order to assess the evolution of the biomarkers over time. Post hoc tests were performed as paired samples, and the Student’s *t*-test and the *p*-value were adjusted using Hommel’s method, with the statistical significance considered to be *p* < 0.05.

Statistical analyses were performed using Statistical Analysis System SAS v9.4 and the SAS Enterprise Guide v8.3 interface.

## 3. Results

### 3.1. Baseline Characteristics of Participants

A total of 278 potential participants were recruited, 177 of whom did not meet the inclusion/exclusion criteria. Therefore, the study population consisted of 101 participants (which aligns with the number in the safety population) with a mean (SD) age (at the time of the study) of 29.8 (7.59) years old; more than half of the study population were women (*n* = 66, 65.35%). Regarding their baseline 25(OH)D levels (Table 1), 94 participants (93%) had 25(OH)D levels from 10 to <20 ng/mL. and seven (7%) had 25(OH)D levels <10 ng/mL.

### 3.2. Efficacy Results

#### 3.2.1. Treatment Phase

During the treatment (open label) phase, participants received either their monthly (*n* = 94) or biweekly (*n* = 7) calcifediol treatment, depending on their baseline 25(OH)D levels (Figure 1). Five out of one-hundred and one subjects were not included in the efficacy analysis (FAS), as they did not meet the criteria established in this population set.

**Figure 2:**
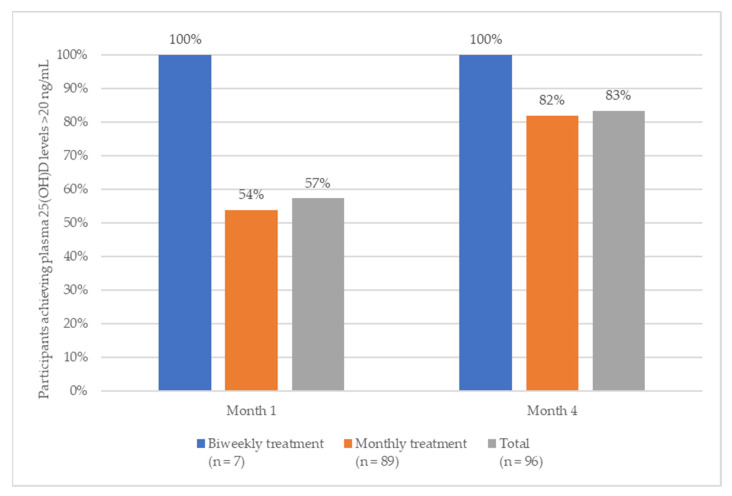
Proportion of participants achieving 25(OH)D levels >20 ng/mL during months one and four.

During month one, 57% of subjects achieved levels that were within the 25(OH)D target range (100% of subjects with biweekly treatment and 54% of subjects with monthly treatments) (Figure 2).

After four months of treatment (primary endpoint), more than 80% of the participants reached the pre-specified 25(OH)D target range (20–60 ng/mL). More specifically, 82% of participants (73 out of 89) who received monthly calcifediol, and 86% of those who received a biweekly treatment (six out of seven) reached the target range (100% of these subjects reached levels above 20 ng/mL, one subject exceeded the upper limit of 60 ng/mL).

By the end of the first month, 8.3% of the subjects had achieved a 25(OH)D level in the range of 30-60 ng/mL, with 28.6% achieving this result in the biweekly treatment group, and 6.7% achieving this result in the monthly treatment group. This percentage notably increased to 45.8% after four months of treatment, with 85.7% in the biweekly treatment group and 42.7% in the monthly treatment group, respectively.

**Figure 3:**
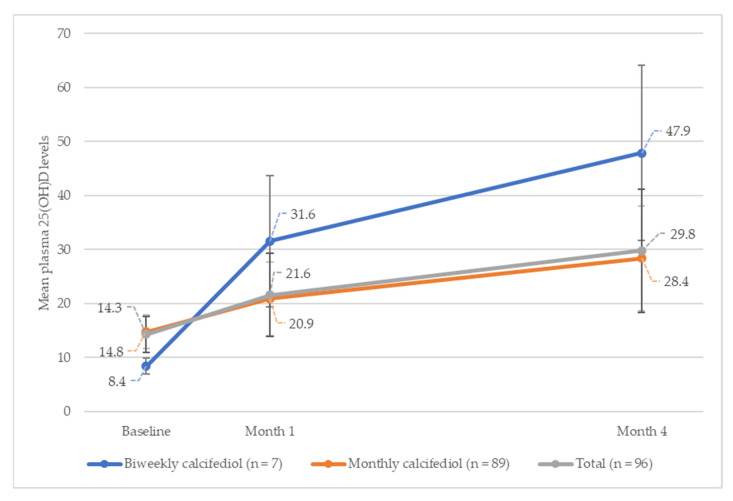
Evolution of plasma 25(OH)D levels, according to the regimen administered (biweekly and monthly) (*n* = 96).

Figure 3 shows the evolution of plasma 25(OH)D levels, at different time points, up to month four, for each treatment group.

In the overall study population, 25(OH)D levels increased from the baseline, by a mean (SD) of 7.3 (8.03) ng/mL during month one, and by 15.5 (12.17) ng/mL during month four (*p* < 0.001) (*n* = 96). When analysed by subgroup, the increase in mean (SD) 25(OH)D levels at month one was 23.2 (11.99) ng/mL for the biweekly treatment group, and 6.1 (6.19) ng/mL for the monthly treatment group, increasing to 39.5 (15.98) ng/mL and 13.7 (9.67) ng/mL, respectively, by month four.

By month four, 16 subjects did not reach their target 25(OH)D levels, and they received two additional months of calcifediol treatment. By month six, 65% of them had levels within the 25(OH)D target range, and the five subjects who did not reach this level were withdrawn from the study.

At the end of the treatment phase (also considering subjects treated until month six), a total of 89 participants (92.7%) had achieved the pre-specified target range (six (86%) who had received biweekly treatments and eighty-three (93.2%) who had received monthly calcifediol therapy) and were randomised for the follow-up blind phase. Two subjects withdrew their consent immediately after randomisation, leaving 87 subjects in the follow up phase.

#### 3.2.2. Follow-Up Phase

During the follow-up phase, 87 participants were chosen to blindly receive monthly treatments of calcifediol (*n* = 43) or placebo (*n* = 44). There were no differences in 25(OH)D levels at the beginning of this period, as follows: 31.1 (8.2) ng/mL and 31.0 (7.5) ng/mL were the levels of subjects given calcifediol and placebo, respectively (Supplementary Table S1). A total of 75 participants (86%) completed the study in accordance with the protocol, having started with severe vitamin D deficiency (*n* = 5) or moderate vitamin D deficiency (*n* = 70).

At the end of the follow-up phase, a significantly higher proportion of subjects who were given calcifediol maintained their 25(OH)D levels within the target range, compared with the placebo group (89% vs. 49%; *p*-value < 0.001).

Plasma 25(OH)D levels were significantly higher in the calcifediol group compared with those in the placebo group (mean [SD]: 25.1 [5.02] ng/mL and 19.1 [6.57] ng/mL, respectively; *p* = 0.000) by the end of the follow-up phase.

**Figure 4:**
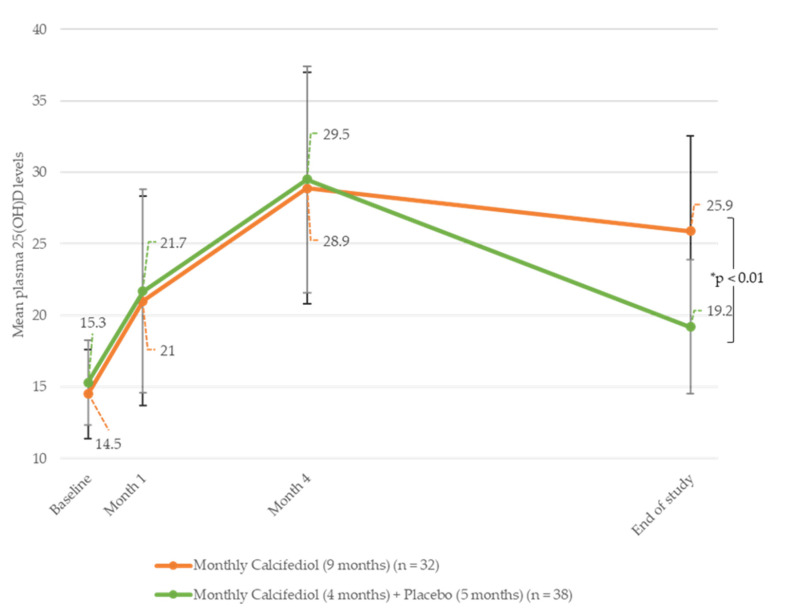
Evolution of the plasma 25(OH)D levels of subjects receiving monthly treatment during the study. Monthly calcifediol treatment during the treatment and follow-up phases (*n* = 32) and monthly calcifediol treatment during the treatment phase, plus monthly placebo given during the follow-up phase (*n* = 38). * *p*-values refer to the comparison, at the end of the study, between the mean 25(OH)D plasma levels of patients receiving calcifediol during treatment and the follow-up phases, and those who received calcifediol in the treatment phase but placebo in the follow-up phase.

The evolution of plasma 25(OH)D levels throughout the study was also analysed using subgroups (Figure 4). We found that subjects receiving monthly calcifediol during both study phases reached steady 25(OH)D levels (28.9 (7.91) ng/mL vs. 25.9 (4.67) ng/mL in the treatment and follow-up phases, respectively) by the end of the study (*n* = 32). However, participants receiving placebo during the follow-up phase experienced a reduction of 25(OH)D levels to 19.2 (6.64) ng/mL (*n* = 38).

Subjects receiving biweekly calcifediol treatment during the treatment phase and monthly calcifediol treatment during the follow-up phase showed a significant reduction in their 25(OH)D levels, from 42.5 (11.27) ng/mL to 19.0 (3.46) ng/mL (*n* = 4). There was only one subject who received biweekly calcifediol treatment during the treatment phase and placebo in the follow-up phase who had reached 25(OH)D levels of 37.0 ng/mL by month four, and they ended the study with levels of 16.0 ng/mL by month nine.

### 3.3. Safety Results

Only one subject presented 25(OH)D plasma levels above 60 ng/mL (78 ng/mL), and they were consequently stopped from receiving calcifediol treatment, in accordance with the protocol. The subject was monitored until the last visit of the study, where their 25(OH)D levels were found to be 10 ng/mL, without any adverse event having occurred.

During the study, 66 AEs were reported by approximately one-third (*n* = 36, 36%) of the safety population (*n* = 101). Of the total AEs, nine (13.64%) were considered to be potentially related to the study treatment; all of them occurred during the treatment phase, and they were reported by five (5%) participants in the group who were being administered calcifediol each month.

All of these AEs, which included headache (one subject), two non-clinically relevant increases in PTH (one subject), nausea (one subject), abdominal discomfort (one subject), decreased appetite (one subject), and three episodes of diarrhoea (two subjects), were mild or moderate in severity, and all participants recovered without complications.

During the study, one participant experienced three AEs (abdominal discomfort, diarrhoea, and decreased appetite) simultaneously, and another reported the same AE twice (diarrhoea episodes).

No serious AEs or AEs leading to discontinuation were reported.

One case of pregnancy was reported during a participant’s final visit. At that time, the participant had already completed the treatment as per the protocol, so no action was taken regarding the study treatment. When the blind test concluded, it was revealed that this participant had been assigned to the placebo group. In addition (outside of the study), the participant was prescribed an additional treatment of oral calcifediol (0.266 mg monthly, for the next 54 days) by her obstetrician due to vitamin D deficiency (<15 ng/mL). In accordance with the protocol, this participant was observed until delivery, which was normal, with no maternal or birth-related complications.

Regarding bone metabolism parameters, only calcium and albumin showed statistical differences between baseline levels and the values reached at month 4 of the participants in the calcifediol monthly treatment arm, with no clinical impact on their health status (Table 2).

## 4. Discussion

This phase I, multicentre trial provides clear evidence of the efficacy and safety of calcifediol 0.266 mg soft capsules for the treatment of vitamin D deficiency (defined as serum 25(OH)D levels <20 ng/mL) in young adults. It also confirms the need to maintain treatment once optimal 25(OH)D levels have been reached, when only pharmacological treatment is used, without a significant change in the patients’ lifestyle.

Previous studies have shown the efficacy and safety of this drug at different dosages in older adults and postmenopausal women [10,13,14,15,16,17]. However, to our knowledge, this is the first study to evaluate the behaviour of calcifediol (testing two different regimens) in a young adult population with vitamin D deficiency and no other associated comorbidities. To that end, two different calcifediol dose regimens were used, as follows: calcifediol 0.266 mg/month for subjects with baseline 25(OH)D levels from 10 to <20 ng/mL; and calcifediol 0.266 mg every two weeks for subjects with baseline 25(OH)D levels <10 ng/mL. These regimens were selected according to clinical practice by Spanish physicians and were also in accordance with the 96 Note of the Italian Medicines Agency (Agenzia Italiana del Farmaco, AIFA). The study comprised two phases, as follows: an open-label phase to evaluate the efficacy of monthly and biweekly treatments with calcifediol (0.266 mg); and a double-blind phase to analyse the fluctuations in stable 25(OH)D levels when treatments with calcifediol 0.266 mg were continued, discontinued, or when the frequency of administration was changed.

In the open-label phase of the study, results showed that monthly treatment with calcifediol (0.266 mg) in young adults for four months raised 25(OH)D levels by 13.7 (9.67) ng/mL from baseline, and biweekly treatments raised them by 39.5 (15.98) ng/mL, with 82% and 100% of the subjects reaching 25(OH)D levels ≥20 ng/mL.

These results are consistent with those from previous studies that used the same dosage of calcifediol in other populations. A previous retrospective observational study, conducted by Olmos et al. (2018), included 156 patients with osteoporosis (23 males and 133 females) who had received monthly or biweekly treatments with calcifediol (0.266 mg), and they reported a significant increase in the concentration of 25(OH)D with both treatment regimens, as follows: monthly calcifediol treatments (0.266 mg/month) produced an increase from a mean (SD) of 23.3 (8.3) ng/mL to 38.8 (12.5) ng/mL, *p* < 0.001, and biweekly calcifediol treatments (0.266 mg/biweekly) produced an increase from a mean (SD) of 16.7 (9.3) ng/mL to 56.2 (18.5) ng/mL; *p* < 0.001 [18]. Similarly, a recent clinical trial, conducted by Pérez-Castrillon et al. (2021), of postmenopausal women showed a mean (SD) 25(OH)D increase of 14.9 (8.1) ng/mL after four months of monthly treatments with calcifediol (0.266 mg (*n* = 200)) [16].

In a study performed by Navarro-Valverde et al. (2016), 40 post-menopausal women with osteopenia (mean age: 67 years) were randomly assigned to four treatment groups; one of them required the participants to take calcifediol (0.266 mg) every two weeks (*n* = 10). The mean (SD) 25(OH)D levels at 0, 6, and 12 months were 39.5 (4) nmol/L (15.8 ng/mL), 164.5 (41.7) nmol/L (65.8 ng/mL), and 210.5 (22.2) nmol/L (84.2 ng/mL), respectively. They found that patients taking a biweekly dose reached 25(OH)D levels above 60 ng/mL by month six, and above 80 ng/mL by month twelve [19]. In the present study, the highest concentration was 78 ng/mL (biweekly group) by month four. The increases in 25(OH)D levels, as described by Navarro-Valverde et al., were compared to those obtained in our study and by the other authors mentioned above; these increases could be explained by differences in analytical methods or procedures.

We also found that when calcifediol treatment is withdrawn, levels decrease below 20 ng/mL. This observation is consistent with the studies performed by Graeff-Armas in 2020 [14] and Pérez-Castrillón in 2023 [17], for daily and monthly calcifediol doses, respectively.

With respect to the potential impact of the changes caused by the calcifediol dosage regimen, four participants in this study initially received biweekly calcifediol doses during the treatment phase, and they achieved the target levels required to progress to the maintenance phase. During the follow-up phase, these participants were randomly assigned to the monthly calcifediol group, and they showed a significant reduction in 25(OH)D levels when the study ended. It is important to note that this situation was observed in only four participants; thus, it is impossible to draw broader conclusions. Further research is therefore necessary.

In our study, no relevant safety issues were detected in relation to the study drug, indicating that calcifediol (0.266 mg) soft capsules, taken monthly and biweekly, are safe dosages for long-term administration.

The main strengths of this study concern the fact that it provided insights into the utilization of calcifediol in the general population, particularly among young adults, thus complementing the existing body of knowledge which predominantly focuses on older age groups. Additionally, the obtained medium- and long-term results contribute to our understanding of calcifediol’s safety profile, even when administered at varying dosages. Moreover, our results contribute to the knowledge on calcifediol usage at different frequencies within the study population, which mimics its usage in clinical practice, and confirm calcifediol’s adaptability to suit the unique needs of individual patients.

On the other hand, a potential limitation of this study concerns the low number of participants recruited and included in the initial biweekly treatment group. This may be due to the lower prevalence of severe vitamin D deficiency (25(OH)D levels <10 ng/mL) in the younger population, which has already been reported to be 7% for the general population [20]. Notably, none of the participants withdrew from the study due to safety concerns, and the statistical analysis revealed strong reliable results.

## 5. Conclusions

In summary, this study provides evidence that calcifediol (0.266 mg) is an effective and safe treatment for young adults with vitamin D deficiency, whether administered on a monthly or biweekly basis; this is consistent with the previously demonstrated efficacy of calcifediol at similar dosages in older adults and postmenopausal women. Notably, biweekly dosages may be particularly beneficial when aiming for more significant increases in 25(OH)D levels, such as in cases of severe vitamin D deficiency or when targeting higher 25(OH)D levels. Nonetheless, due to the small sample size in the biweekly dose group, further studies are needed to confirm these results. This study also underscores the necessity of ongoing treatment maintenance even after achieving optimal 25(OH)D levels, suggesting that long-term treatment may be of use in cases of vitamin D deficiency. Ultimately, it reinforces the safety of calcifediol (0.266 mg) soft capsules being taken on a monthly and biweekly long-term basis.

## Figures and Tables

**Table 1:** Baseline characteristics of the study population, according to their baseline plasma 25(OH)D levels.

Variable	Biweekly Treatment, *n* = 7	Monthly Treatment, *n* = 94	Total, *n* = 101	*p*-Value
Age, *years*				
Mean (SD)	32.7 (7.95)	29.5 (7.56)	29.8 (7.59)	0.205 ^a^
Median (Q1; Q3)	29.0 (25.0; 41.0)	29.0 (24.0; 32.0)	29.0 (24.0; 34.0)	
Sex, *n* (%)				
Male	1 (14.29)	34 (36.17)	35 (34.65)	0.417 ^b^
Female	6 (85.71)	60 (63.83)	66 (65.35)	
Race, *n* (%)				
Caucasian	4 (57.14)	77 (81.91)	81 (80.20)	0.026 ^b^
Asian	0 (0.00)	1 (1.06)	1 (0.99)	
Black	0 (0.00)	0 (0.00)	0 (0.00)	
Hispanic	2 (28.57)	15 (15.96)	17 (16.83)	
Other	1 (14.29)	1 (1.06)	2 (1.98)	
BMI, kg/m^2^				
Mean (SD)	22.8 (1.68)	23.9 (3.06)	23.8 (2.99)	0.387 ^c^
Median (Q1; Q3)	22.9 (21.0; 24.5)	23.7 (21.5; 26.4)	23.6 (21.5; 26.3)	

^a^ Mann–Whitney; ^b^ Fisher’s test; ^c^ Student’s *t*-test. BMI: Body Mass Index; Q: Quartile; SD: Standard deviation.

**Table 2:** Evolution of the biochemical parameters of bone and mineral metabolism during the study.

**Change from Baseline to End of the Treatment Phase**				
	**Biweekly Calcifediol (*n* = 7)**		**Monthly Calcifediol (*n* = 89)**	
**Variable**	**Mean (SD)**	***p*** **-Value**	**Mean (SD)**	***p*** **-Value**
*25(OH)D*	39.5 (15.98)	0.001 ^a^	15.4 (9.39)	>0.001 ^b^
*PTH*	−3.0 (31.05)	0.807 ^a^	−2.8 (20.66)	0.208 ^a^
*ALP*	−6.4 (10.47)	0.155 ^a^	1.8 (8.26)	0.044 ^a^
*Albumin*	−0.4 (0.40)	0.038 ^a^	−0.1 (0.23)	<0.001 ^b^
*Calcium*	0.1 (0.33)	0.336 ^a^	0.2 (0.37)	<0.001 ^b^
*Phosphate*	0.2 (0.39)	0.329 ^a^	−0.0 (0.55)	0.602 ^a^
**Change from** **End of Treatment Phase to End of Follow-Up Phase**				
	**Calcifediol (*n* = 36)**		**Placebo (*n* = 39)**	
**Variable**	**Mean (SD)**	***p*** **-Value**	**Mean (SD)**	***p*** **-Value**
*25(OH)D*	−6.0 (11.02)	0.003 ^b^	−10.9 (9.25)	<0.001 ^a^
*PTH*	3.8 (17.78)	0.205 ^a^	6.1 (19.77)	0.064 ^a^
*ALP*	0.9 (9.35)	0.549 ^a^	−0.2 (8.95)	0.901 ^a^
*Albumin*	0.0 (0.24)	0.398 ^b^	−0.0 (0.27)	0.485 ^b^
*Calcium*	0.0 (0.43)	0.877 ^a^	−0.0 (0.32)	0.766 ^a^
*Phosphate*	0.0 (0.54)	0.626 ^a^	0.1 (0.51)	0.305 ^a^

Units: 25(OH)D, ng/mL; Albumin, g/dL; ALP, IU/L; Calcium, mg/dL; Phosphate, mg/dL; PTH, pg/mL. ^a^ Student’s *t* test; ^b^ Wilcoxon signed–rank test.

## Data Availability

The data presented in this study are available on request to the corresponding author. The data are not publicly available due to confidentiality issues.

## Supplementary Materials

The following supporting information can be downloaded at: https://www.mdpi.com/article/10.3390/nu16020306/s1, Table S1: Baseline characteristics of the population who completed the treatment phase.
