# Supplementary material for: Efficacy and Safety of Calcifediol in Young Adults with Vitamin D Deficiency: A Phase I, Multicentre, Clinical Trial—POSCAL Study
Source: Nutrients. 2024 Jan 19;16(2):306. doi: 10.3390/nu16020306 (PMC10818887; doi:10.3390/nu16020306)
Supplement: Supplementary file 1 [file nutrients-16-00306-s001.zip › nutrients-2813244-supplementary.pdf]

Table S1. Baseline characteristics of the population who completed the treatment phase.

Variable	Placebo (*n* = 45)	Calcifediol (*n* = 44)	Total (*n* = 89)	*p-value*
Age (years)				0.301[a]
N	45	44	89	
Mean (SD)	30.0 (7.86)	29.2 (7.38)	29.6 (7.59)	
Median (Q1; Q3)	30.0 (24.0; 32.0)	29.0 (24.5; 32.5)	29.0 (24.0; 32.0)	
Sex, n (%)				0.155[b]
N	45	44	89	
Male	12 (26.67)	18 (40.91)	30 (33.71)	
Female	33 (73.33)	26 (59.09)	59 (66.29)	
Race, n (%)				0.042[c]
N	45	44	89	
Caucasian	37 (82.22)	35 (79.55)	72 (80.90)	
Asian	1 (2.22)	0 (0.00)	1 (1.12)	
Black	0 (0.00)	0 (0.00)	0 (0.00)	
Hispanic or Latino	6 (13.33)	9 (20.45)	15 (16.85)	
Other	1 (2.22)	0 (0.00)	1 (1.12)	
Body mass index (kg/m^2)				0.319[d]
N	45	43	88	
Mean (SD)	24.1 (2.96)	23.5 (3.08)	23.8 (3.02)	
Median (Q1; Q3)	23.9 (22.2;26.4)	22.9 (20.9;25.7)	23.6 (21.5;26.4)	
Min; Max	19;30	19;29	19;30	

[a] Mann-Whitney; [b] Chi-square test, [c] Fisher's test; [d] Student's T-test. Q: Quartile; SD: Standard deviation.
